# What Makes Cirrhosis Irreversible?—Consideration on Structural Changes

**DOI:** 10.3389/fmed.2022.876293

**Published:** 2022-04-27

**Authors:** Katalin Dezső, Sándor Paku, László Kóbori, Snorri S. Thorgeirsson, Péter Nagy

**Affiliations:** ^1^Department of Pathology and Experimental Cancer Research, Semmelweis University, Budapest, Hungary; ^2^Department of Surgery, Transplantation and Gastroenterology, Semmelweis University, Budapest, Hungary; ^3^Laboratory of Human Carcinogenesis, Center for Cancer Research (CCR), National Cancer Institute, National Institute of Health (NIH), Bethesda, MD, United States

**Keywords:** liver regeneration, parenchymal extinction, budding, progenitor cells, ductular reaction

## Abstract

Several studies have shown that liver fibrosis, and even cirrhosis can be reversed, disproving the old “dogma” that cirrhosis is irreversible. In addition to scaring, vascular alterations appear to be critically important in the progression of chronic liver diseases. To overcome the “tipping-point” of cirrhosis, we need to understand in depth what might make it irreversible in some cases. Morphologically, the initial, as well as the advanced stages of cirrhosis are characterized by specific structural changes. The hallmark of the initial stage is the division of the original liver parenchyma by centro-central or porto-portal septa. No significant vascular changes are observed in this stage. The advanced stage is characterized by several morphological alterations: (i) The main feature is the parenchymal extinction, with intact portal vein branches, hepatic artery branches, and biliary ductules; (ii) In the extinct areas we observed numerous loops in the ductular network, indicating the disruption of the hepato-biliary junctions; (iii) Although the ductular progenitor cells are able to generate hepatocytes *via* the budding process, the newly formed hepatocyte nodules cannot re-establish the original lobular architecture due to their disorganized growth. In conclusion, this regenerative process characteristic for the advanced stage, contributes to circulatory disorders, perpetuates parenchymal injury and may lead to the irreversibility of cirrhosis.

Cirrhosis is one of the oldest terms in hepatology, coined by Laennec (1) more than 250 years ago, but it was inaccurate from the beginning.

The term is derived from the Greek kirrhos, “yellow,” however, it is not yellowness, but progressive fibrosis and vascular changes that define cirrhosis. By definition, cirrhosis means the distortion of the hepatic architecture by dense fibrotic bands and nodules of hepatocytes.

In addition, severe vascular alterations (capillarization of the sinusoids and the formation of various shunts) are also typical features of this process. The clonal nature of the nodules, as well as the lack of common mutations between adjacent nodules, raised the possibility that these are not entirely the result of a simple division of the liver parenchyma (2, 3).

Cirrhosis was generally regarded as the final, common, irreversible stage of chronic liver diseases. However, recent evidences have started to undermine this dogma (4–6). In most experimental models, although it is debatable whether fibrosis or cirrhosis develops, the connective tissue disappears rapidly after the cessation of the hepatic insult. However, once regenerative nodules have appeared, the progression is irreversible (7).

Reliable reports were published about the regression or disappearance of fibrosis in different human liver diseases (8–11). Wanless reported a continuum of regressive changes in fibrotic/cirrhotic livers, resulting in an almost normal hepatic parenchyma (12). These observations have given rise to intense debate about the irreversibility of cirrhosis. The outcome is now almost definitively accepted: cirrhosis can regress, and a panel of distinguished hematopathologists proposed that the term of cirrhosis should be discontinued (13).

Unfortunately, the terms cirrhosis/fibrosis or reversion/regression are not used consistently, which further hinders the clarification of the terminology. Desmet and Roskams (8) thoroughly discuss the limitations of studies on the reversibility of cirrhosis and conclude that “architectural distortion and even more the vascular shunts in portal–central septa and in larger fibrous scars of multinodular parenchymal extinction, are of such slow reversibility that—from the point of view of expected remaining life span of the patient—these lesions are for all practical purposes irreversible.”

Hytiroglou and Theise (14) commemorating Wanless's milestone publication (12) also claim that “many vascular lesions of cirrhotic livers are not thought to regress” and “regression of cirrhosis does not imply regression of molecular changes.” They also indicate that some form of permanent damage may develop in the chronically injured hepatic tissue.

Direct acting antiviral treatment (DAA) revolutionized the treatment of HBV and HCV infected patients. There is no doubt that the elimination of the virus reverses early stages of hepatic fibrosis. Moreover, there seems to be a general agreement that not even sustained virological response results in the regression of advanced cirrhosis and its related complications (15–17). Iron reduction therapy resulted in fibrosis stage improvement only in a portion of hemochromatosis related liver disease (18). Clinical experience has shown that cirrhosis is often irreversible (19) and cannot be reversed by the removal of the causative agent.

Cirrhosis induced in rodents by repeated administration of fibrogenic compounds [carbon tetrachloride (CCl_4_) or thioacetamide (TAA)] is mostly reversible, but prolonged CCl_4_ or TAA treatment results in so severe architectural changes of the liver, that the rats show all features of decompensated cirrhosis without any sign of recovery (20).

Despite widely accepted thought on end-stage liver disease, it is important to assess if all forms of cirrhosis are reversible.

It is also debatable whether there is a point of no return (i.e., the existence of some form/level of parenchymal and vascular remodeling), which rules out recovery. Beside its academic importance, the determination of such an irreversible stage would have significant clinical implications.

Hepatic venous pressure gradient (HVPG) is probably the most informative parameter, which encapsulates the interplay of several factors in a single value, and it also provides a reliable prognostic forecast for cirrhotic patients (21). HVPG correlates with several morphological parameters and with the histological subclassification of cirrhosis (21–24). Pretreatment values of HVPG predict the histological response to DAA therapy. Patients with high (>14 mm Hg) HVPG value did not show any signs of regression even after SVR (25). Following this parameter may help to illuminate potential causes/mechanisms of irreversibility. HVPG is an indirect indicator of portal pressure, which is determined by extrahepatic and hepatic parameters. The most important extrahepatic parameters, such as splanchnic hyperemia, and the development of porto-systemic shunts, are probably not related to the reversibility of hepatic architectural distortions. The increased intrahepatic resistance is also the summation of several factors [e.g., fibrous tissue deposition, endothelial dysfunction, thrombosis, intrahepatic vascular shunts between afferent and efferent vessels (21)], but the most important component seems to be the distortion and compression of the efferent venous system (22). Mitra (26) in his seminal work studied the vascular alterations of cirrhotic livers with simultaneous injection of India ink and carmine gelatin. The lesions were substantially the same in experimental and human advanced cirrhotic samples. When compared with the portal veins, the hepatic veins were markedly flattened. In agreement with previous investigations, he concluded that the hepatic venous compression or luminal obstruction appears to be the chief cause of portal hypertension (27, 28).

Both experimental and human liver cirrhosis set in by the formation of centro-central porto-portal, and/or porto-central fibrotic bands. These septa split the pre-existent parenchyma without altering the vascular architecture of the liver. Later, hepatic nodules separated by fibrotic bands will characterize the liver (Figures 1A,B). This condition is already called cirrhosis. Such cirrhotic livers are often seen in experimental models, which can rapidly regress upon the withdrawal of the toxic compounds. Portal triads, as well as hepatic veins can be easily recognized in these specimens and no compressed hepatic veins can be seen. Since there is no essential distortion of architecture and it is completely reversible, Quinn and Higginson (7) proposed that the formation of centro-central septa would be better termed as “reversed lobulation.” As this form of cirrhosis in humans probably does not result in symptoms, such specimens are rarely seen by clinical pathologists.

**Figure 1:**
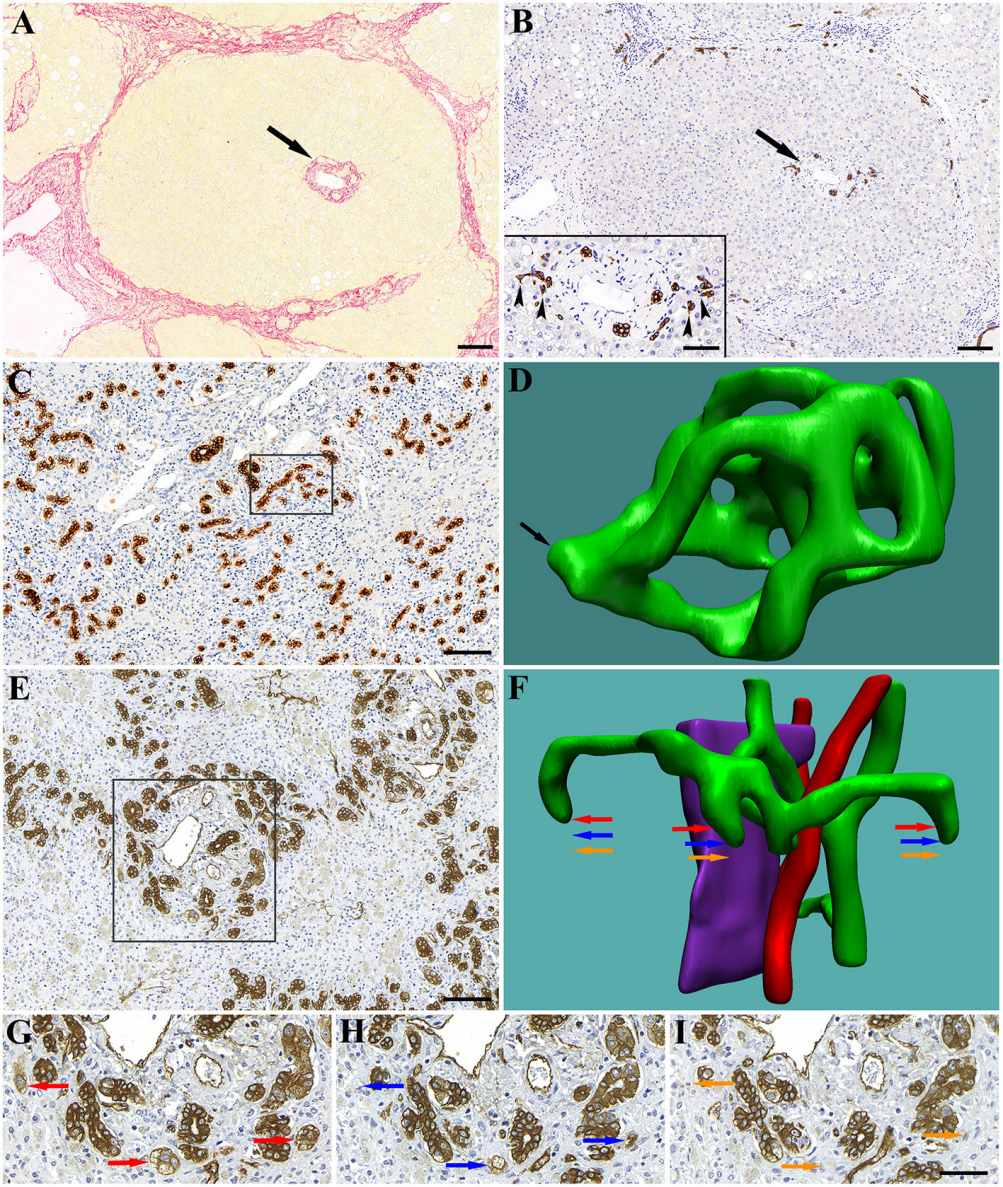
Early (first) stage of human liver cirrhosis. The liver is composed of portal lobules with intact portal triads in the center (arrows). **(A)** Picrosirius-red staining highlights the connective tissue surrounding the portal lobule. **(B)** CK7 staining shows the biliary structures in the portal area. Inset shows the spiky shape of bile ductules (canals of Hering) terminating on hepatocytes (arrowheads). Advanced (second) stage of human liver cirrhosis. In the extinct areas the ductules proliferate (by a process of ductular division) and form a dense network composed of numerous loops. **(C)** Low magnification of a CK7 stained extinct area. A high number of rounded cross-sections of bile ductules and no hepatocytes are visible. **(D)** 3D reconstruction of 20 serial sections (boxed area on **C**) reveals numerous loops in the ductular network within this small tissue volume. Arrow points at a section of the network through which it is connected to the ductular tree (not reconstructed). Massive hepatic necrosis in human liver is characterized by parenchymal extinction (collapse) and numerous blind-ended, rounded ductules around the portal areas. **(E)** Low magnification of a CK7 stained extinct area. A high number of rounded cross-sections of bile ductules is visible. **(F)** 3D reconstruction of a portal area (boxed area on **E**). Fifty-one serial sections were used for the reconstruction. Green, bile ductules; Pink, portal vein; Red, arteries. **(G–I)** High power micrographs of representative serial sections showing the blind ends of ductules. Areas marked by colored arrows show their position on the reconstruction **(F)**. Scale bar: **(A–C,E)** 100 μm; Inset and **(G–I)** 50 μm.

We have proposed to stratify human cirrhosis into two stages (29) and the above mentioned changes would characterize the early stage. The hallmark of the advanced stage is parenchymal extinction and the formation of hepatic nodules in these areas by a “budding” process of the biliary ductules, as recognized and described by Stueck and Wanless (30).

The bile ductules in the healthy human liver show ramifications with a tree-like appearance terminating on hepatic plates. Occasionally, loops can be present, but no blind ends or complex network were seen (data not shown). In contrast, in the extinct areas of cirrhotic livers, a ductular network composed of numerous tortuous loops was observed, most likely generated by an intussusceptive-like process (Figures 1C,D). Similar bile ductule morphology was detected in the liver samples with massive necrosis, where the terminations of the biliary ductular system were also sealed (Figures 1E–I).

Either way, the progenitor cells, residents of the ductules, lost contact with the hepatic plates, but still possess regenerative capacity and are able to respond to the so far undefined hepatic functional failure by budding.

The careful morphological analysis of such advanced human and experimental cirrhotic samples revealed the presence of portal vein branches in the center of the nodules, while compressed flattened central veins were consistently outside, situated in the connective tissue (29). These vessels were crushed by the expanding cirrhotic nodules. In addition, the sinusoids inside these nodules were usually dilated, reflecting the obstructed efferent flow. Moreover, comparative clinico-pathological observations indicated that nodular regeneration in the cirrhotic liver is very frequently associated with portal hypertension (31).

In the livers of a subgroup of patients with fulminant hepatic failure, hepatic nodules with almost identical architectural arrangement could be observed (portal venules in the center of the hepatic nodules). In these regenerating livers, the hepatic nodules located around the compressed hepatic veins, can be considered as an attempt to reconstruct the hepatic lobule (32). If the patient survives without transplantation, this pattern of regeneration would end up in “post-necrotic macronodular” cirrhosis (31, 33), another irreversible condition.

Wanless (34) emphasizes the role of vascular injury in the pathogenesis of parenchymal extinction. The compressed hepatic veins may initiate a vicious circle of parenchymal extinction, budding and compression of hepatic veins.

The progenitor cells (called oval cells in rat) are able to regenerate the normal hepatic parenchyma (35), but at the ductular-parenchymal junction there is a fundamental difference between the oval cell mediated regeneration and “budding” process. The natural hepatobiliary junction, outlined by an open or U shaped termination of the ductular basement membrane on the hepatic plate, is constantly preserved in the 2-acetylaminofluorene/partial hepatectomy model. However, in advanced cirrhotic or in necrotic livers this junction is disrupted by parenchymal extinction. Following the extinction in cirrhotic or necrotic livers, these architectural elements are distorted by the proliferating myofibroblasts, also preventing the reestablishment of hepatic lobules from the emerging hepatocytes. Thus, in the absence of a proper architectural frame (basement membrane scaffold), this process will only result in a frustrated regenerative reaction, characterized by the production of individual regenerative nodules, unable to regenerate normal hepatic structures. Moreover, the expanding regenerative nodules compress the low pressure efferent vessels (hepatic veins) inflicting parenchymal congestion, necrosis/scarring and a new cycle of regeneration. This self-supporting process impedes proper liver regeneration, and even in the absence of the original liver damaging insults results in end-stage, irreversible liver disease (Figure 2).

**Figure 2:**
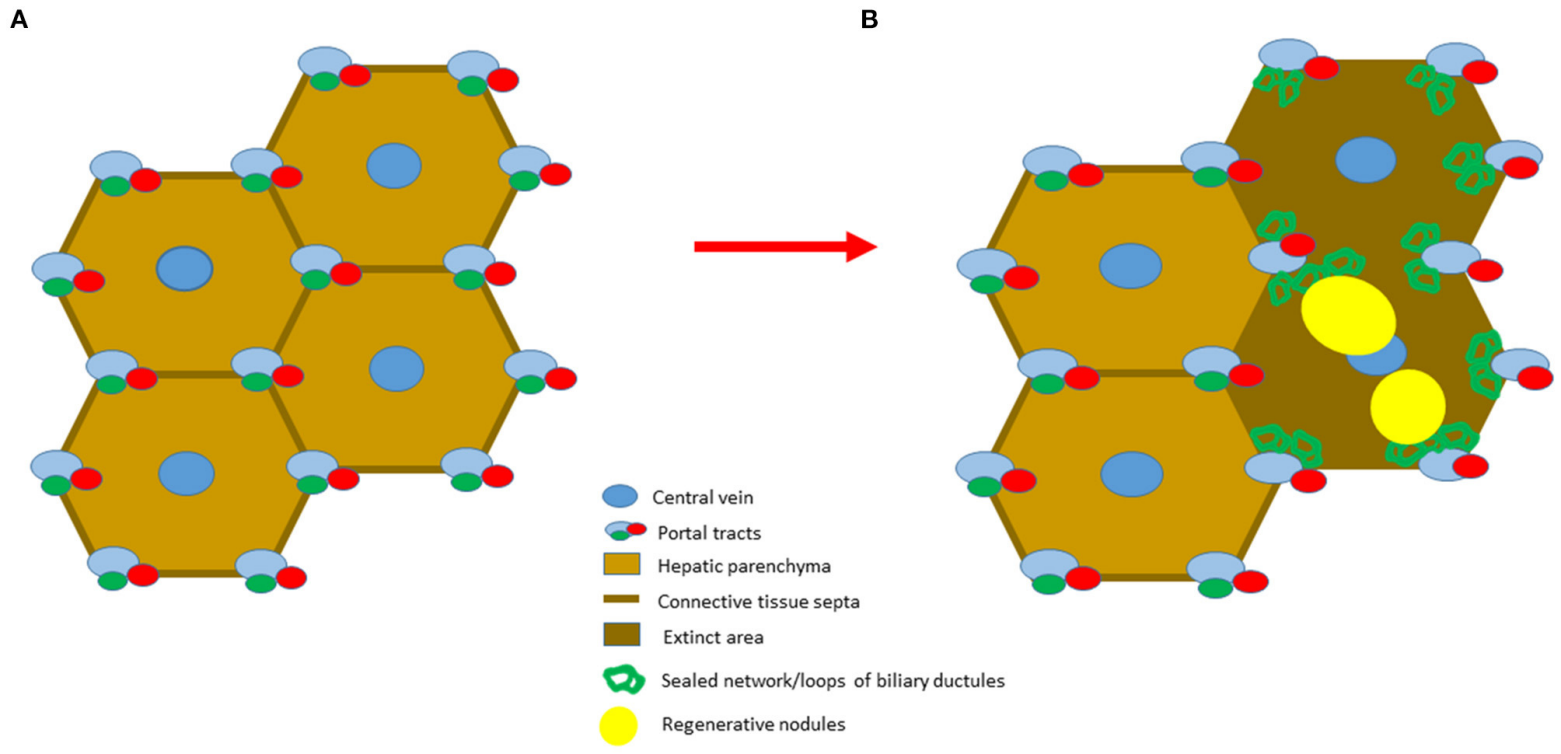
Schematic representation of the two stages of cirrhosis. **(A)** The initial stage is characterized by a simple division of hepatic parenchyma by fibrotic septa. **(B)** The advanced stage of cirrhosis is characterized by the presence of parenchymal extinction, where preserved portal vein, hepatic artery and hepatic vein branches are present. The loss of hepatobiliary junctions leads to the distortion of the biliary ductules. As a consequence, the newly formed regenerative nodules are unable to restore the original lobular architecture due to their disorganized growth.

## Data Availability Statement

The raw data supporting the conclusions of this article will be made available by the authors, without undue reservation.

## Ethics Statement

The studies involving human participants were reviewed and approved by the study protocol was approved by the Ethical Commission of Semmelweis University (No. 125/2010). Written informed consent for participation was not required for this study in accordance with the national legislation and the institutional requirements.

## Author Contributions

PN was responsible for the concept. PN, KD, and SP were responsible for preparing the manuscript. LK, KD, SP, and PN were responsible for the literature review. The immunohistochemical stainings were effectuated by KD. SP was responsible for the 3D reconstruction. ST critically revised the article. All authors reviewed the manuscript prior to submission and contributed to the article and approved the submitted version.

## Funding

This work was supported by NKFIH-FK 138673 (KD).

## Conflict of Interest

The authors declare that the research was conducted in the absence of any commercial or financial relationships that could be construed as a potential conflict of interest.

## Publisher's Note

All claims expressed in this article are solely those of the authors and do not necessarily represent those of their affiliated organizations, or those of the publisher, the editors and the reviewers. Any product that may be evaluated in this article, or claim that may be made by its manufacturer, is not guaranteed or endorsed by the publisher.

## Abbreviations

DAA: direct acting antiviral treatment
HBV: hepatitis B virus
HCV: hepatitis C virus
CCl_4_: carbon-tetrachloride
TAA: thioacetamide
HVPG: hepatic venous pressure gradient
SVR: sustained virologic response.
